# Explaining variance in breastfeeding intentions and behaviors among a cohort of Midwest mothers using a theory of planned behavior-based structural model

**DOI:** 10.1186/s12884-022-04628-9

**Published:** 2022-04-13

**Authors:** Anita Esquerra-Zwiers, Emilie Dykstra Goris, Aaron Franzen

**Affiliations:** 1 Department of Nursing, Hope College, Holland, USA; 2 Department of Sociology, Hope College, Holland, MI USA

**Keywords:** Breast feeding, Intention, Social norms, Models, theoretical, Structural equation model

## Abstract

**Background:**

The Theory of Planned Behavior (TPB) has guided the investigation of breastfeeding since the 1980’s, incorporating the major constructs of attitudes, subjective norms/normative beliefs, perceived behavioral control, and intentions*.* The purpose of this research study was to define a TPB-based structural latent variable model so as to explain variance in breastfeeding intentions and behaviors among a cohort of Midwest breastfeeding mothers.

**Methods:**

The longitudinal descriptive study utilized questionnaire data collected from a convenience sample of 100 women with low-risk pregnancies with the intention to breastfeed at three separate time points (> 30 weeks antepartum, 10 and 60 days postpartum). Data were coded and analyzed using IBM SPSS, SAS and the *lavaan* package in R.

**Results:**

Participants were predominantly White (94%, *n* = 94), married (95%, *n* = 95), college-educated (96%, *n* = 96), and had previous breastfeeding experience (75%, *n* = 75). The majority gave birth vaginally (79%, *n* = 75). Varimax analysis revealed a plurality of factors within each domain. Attempts to fit a structural model, including both hierarchical and bi-factor latent variables, failed, revealing a lack of statistical significance and poor fit statistics.

**Conclusion(s):**

These findings illustrate the importance of using methods that fit the phenomena explained. Contributors to poor model fit may include outdated tools lacking cultural relevance, a change in social norms, or a failure to capture the possible influence of social media and formula marketing on breastfeeding behaviors. The null finding is a significant finding, indicating the need to revisit and refine the operationalization and conceptual underpinnings of the TPB through qualitative methods such as exploring the lived experiences of breastfeeding women in the Midwest region.

## Background

The Healthy People 2030 targets [1] indicate a persistent deficit in the United States’ breastfeeding trends, which suggest a need for comprehensive, holistic interventions targeting social, attitudinal, behavioral, and biological factors associated with breastfeeding. Investigations of the social, attitudinal, and behavioral determinants of breastfeeding have been guided by the Theory of Planned Behavior (TPB) since the 1980s [2]. Primary constructs of the TPB include attitudes, subjective norms/normative beliefs, perceived behavioral control, and intentions toward a specified behavior [3]. When employed in breastfeeding research, the *behavior* is breastfeeding, while *intentions* are based on the duration and degree of certainty (exclusive or partial) about carrying out the breastfeeding behavior [2]. The *perceived behavioral control* construct encompasses the degree of expected ease or difficulty with breastfeeding, a perception derived from the mother’s confidence in her ability to achieve breastfeeding expectations based on past or anticipated experiences [4]. *Attitudes* include the intensity of the mother’s beliefs about the possible health effects of breastfeeding including consequences or benefits and risks of breastfeeding for both her and her infant’s health, often defined by breastfeeding knowledge [2]. *Subjective norms/normalization beliefs* encompass the level of social pressures that mothers feel from family, friends, health professionals, and others, toward breastfeeding [4].

Three recently published reviews examined observational studies [5], interventional studies [6], and meta-analysis with structural equation modeling [7] related to the TPB. Lau and colleagues [5] identified an unmet need to explore the TPB in relationship to breastfeeding behaviors. They demonstrated a lack of evidence for a positive relationship between breastfeeding duration and maternal attitudes, subjective norms, or perceived behavioral control. Lau and colleagues [5] provided evidence that new studies are necessary to examine the relationship between breastfeeding motivation and duration. Bai and colleagues [6] critical review examined 18 theoretical-based interventional studies published between January 2008 and December 2018, with five studies specifically using TPB. A few of the TPB studies reviewed by Bai and colleagues [6] examined studies using regression or multivariate analysis, but none analyzed TPB constructs using structural equation modeling (SEM). Gou and colleagues [7] pooled and analyzed data from 10 studies to determine the applicability and efficacy of the TPB in predicting breastfeeding. Their findings suggested a moderate relationship between intention and TPB constructs. These reviews, however, overlooked limitations of tools used to measure norms, attitudes, and perceived behavioral control as related to breastfeeding behaviors and the need to build SEM models that include observed variables for modeling latent variables and not just a correlation matrix. In order to strengthen the evidence to support the TPB, the operational definitions of model antecedents must be carefully examined.

Human milk reduces health complications of the mother and infant. Multiple health organizations recommend human milk as the preferred exclusive infant nutrition for the first 6 months of age [8–10]. Although research supports the benefits of breastfeeding, the 6-month exclusive breastfeeding rates of Michigan infants born in 2015 were 26.6%, with an affiliated 78 average Maternity Practices in Infant Nutrition and Care score [11]. Although these metrics were on par with the national average at the time, the exclusivity rate remains below the 100% recommended by professional health organizations.

Since nationwide, exclusive 6-month breastfeeding rates continue to remain low, this team sought to explore attitudinal, social, perceived behavioral control, and biological factors that may influence maternal perceptions of insufficient milk supply. As part of the larger study, the TBP was employed to examine the maternal perceived attitudinal, social, and perceived behavioral control factors that may contribute to the decision to breastfeed in the Midwest. Therefore, the purpose of this research study was to define a TPB-based structural latent variable model so as to explain variance in breastfeeding intentions and behaviors among a cohort of Midwest breastfeeding mothers.

## Methods

### Design

In order to define a TPB-based structural latent variable model explaining variance in breastfeeding intentions and behaviors, this research team employed a longitudinal descriptive design. Questionnaire data were obtained using a modified version of Manstead’s reconstructed Predictive [Breastfeeding] Questionnaire [2].

### Setting

Data were collected in the Midwest region of the United States as part of a larger Mother’s Milk for Michigan Infants project from April 2018–February 2019 funded by the International Society for Research in Human Milk and Lactation and the Family Larsson-Rosenquist Foundation Trainee Travel Fund [to AEZ]. The larger study used volunteer and paid undergraduate nursing research students to recruit participants and collect both survey data and human milk samples. The study was approved by Hope College’s Human Subjects Review Board. All enrolled participants were provided written research materials and consented prior to participation in the study.

### Sample

One hundred women with low-risk pregnancies (no history of diabetes, chronic hypertension, or known infant congenital anomalies) and the intention to breastfeed were enrolled via convenience sampling after 30 weeks gestation and completed three questionnaires (antepartum, 10 and 60 days postpartum). Women were eligible to participate if they were 21 years of age or older, English proficient, intended to breastfeed with a singleton gestation, and lived within a 75-mile radius of the study site. To reach a large group of mothers intending to breastfeed, the sample was recruited via social media, recruitment materials posted at local hospitals and businesses, and snowballing. Participants were provided with a $20 USD store card as partial compensation for their time and participation.

### Measurement

#### Antepartum questionnaire

The Antepartum Questionnaire collected participant demographic data and included the Predictive Breastfeeding questions developed by Manstead and colleagues [2] (see Table 2) using the TPB. Questions were reviewed for consistency with previously published breastfeeding studies using similar questionnaire data [12, 13]. Participant demographic information included the maternal date of birth, marital status, annual household income, insurance type, the highest level of education, current employment status, race/ethnicity, and employment plans after the baby’s birth. The Predictive Breastfeeding questions examined participants’ attitudes, beliefs, social norms regarding breastfeeding, and behavior using a series of Likert-style questions. Breastfeeding intentions were measured using the Infant Feeding Intentions Scale [14, 15]. Additional questions were included to address perceived behavioral control. The median duration to complete the Antepartum Questionnaire was 13.4 min (first and third quartile: 9.63, 17.52).

#### Day 10 & day 60 questionnaires

The targeted behaviors were exclusive breastfeeding at Day 10 and Day 60 postpartum. The Day 10 and Day 60 Questionnaires measured participants’ feeding practices postpartum. A series of multiple-choice questions measured feeding method, mode of milk expression, and frequency of feeding to conceptualize exclusivity and duration of breastfeeding (see Table 2). The median durations to complete the Day 10 and Day 60 Questionnaires were 7.2 min (4.92, 11.72) and 5.1 min (3.03, 14.93), respectively.

### Data collection

The consent form and questionnaires were administered electronically via Qualtrics^XM^_(R)._ Eligible participants were invited to review and complete an online consent form. Once the consent form was signed, participants were directed to complete the Antepartum Questionnaire. Participants were instructed to notify the research team when they gave birth. Based on the provided birth date, Day 10 and Day 60 Questionnaires were scheduled to be distributed to participants. No more than two reminders to complete any of the three questionnaires were sent to participants.

### Data analysis

**Fig. 1:**
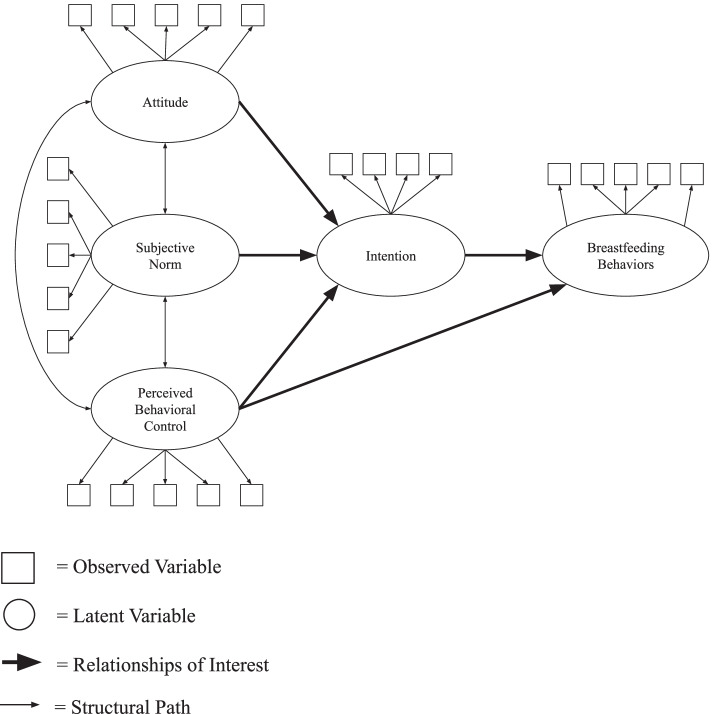
Theory of planned behavior and breastfeeding behavior structural model

Descriptive data were analyzed using IBM SPSS (Version 24). Additional data analysis were completed using SAS University Edition and the *lavaan* package in *R* for latent variable modeling [16]. Analyses included confirmatory factor analysis, exploratory factor analysis including minimum average partial (MAP), very simple structure (VSS), parallels, and varimax rotation for the factor analysis. Finally, structural equation models (SEM) were utilized. MAP, VSS, and parallels provide the researcher a sense of how many factors may be present within any given set of data if one does not know how many should be present. A varimax factor rotation assumes that the different factors are not correlated with one another, thereby decreasing the possibility of a manifest variable strongly loading on more than one factor domain. The strategy was to begin with confirmatory analysis, fit a structural model, and then trim the model for best fit (See Fig. 1).

When it was clear confirmatory factor analysis was insufficient, the strategy became exploratory factor analysis, fit latent variables (LVs) and trim manifest measures if necessary, and fit a structural model. Authors primarily relied on the Bayesian information criterion (BIC) to compare model fit between the first-order LV, hierarchical LV, and bifactor LV (described in more detail below). Model fit for each latent construct was assessed using cutoffs suggested by Schreiber and colleagues [17] and the joint criteria suggested by Hu and Bentler [18]. Schreiber and colleagues [17] suggest a Comparative Fit Index (CFI) of ≥ .95, Tucker-Lewis Index (TLI) of ≥ .95, a Root Mean Squared Error of Approximation (RMSEA) of ≤ .06, and Standardized Root Mean Squared Residual (SRMR) of ≤ .08, and Hu and Bentler [18] suggest either CFI ≥ .96 and SRMR ≤ .09 or SRMR ≤ .09 and RMSEA ≤ .06. Scale reliability was assessed using McDonald’s omega scores instead of alpha scores [19–21], and any missing data were handled with full information maximum likelihood in the structural models [22, 23].

## Results

From the original 100 participants, 87 completed all three questionnaires in full. Participants were predominantly White (94%, *n* = 94), married (95%, *n* = 95), college-educated (96%, *n* = 96), and had previous breastfeeding experience (75%, *n* = 75). The majority gave birth vaginally (79%, *n* = 75). Complete sample characteristics are available in Table 1.

**Table 1:** Sample characteristics

Characteristic	*N* = 100	
	Mean	SD
Maternal age	31.3	3.44
Gestational age-weeks	39.1	1.29
	No.	%
Race/ethnicity, White	94	94
Married	95	95
College educated	96	96
Household income > $75,000	55	55
Private insurance	84	84
Current employment status, Full-time	41	41
Primiparous	23	23
Previous breastfeeding experience	75	75
Vaginal birth^a^	75	78.9
Intention: Exclusively breastfeed at 1 month, Extremely likely	79	79
Intention: Exclusively breastfeed at 3 months, Extremely likely	80	80
Intention: Exclusively breastfeed at 6 months. Extremely likely	69	69
Was the mother providing any breast milk at Day 10^a^	93	97.9
Was the mother providing exclusive breast milk at Day 10^a^	89	93.7
Was the mother providing any breast milk at Day 60^b^	81	94.2
Mother providing exclusive breast milk Day 60^b^	76	87.4

^a^*N* = 95, ^b^*N* = 87

While the study aimed to define a TPB-based structural latent variable model explaining variance in breastfeeding intentions and behaviors among a cohort of Midwest breastfeeding mothers, this did not fit the data. The theoretical model from Duckett and colleagues [4] was not a good fit, as many of the observed variables were not significantly related to one another within constructs in the confirmatory analysis (See ω and α in Table 2 and fit statistics in Table 3).

**Table 2:** Factor loadings and omega/alpha scores

Factor		Description of Item	Standardized Factor Loading (ω, α)
Attitudes	(Q)	*Response Options*: Strongly Disagree (1) to Strongly Agree (7)	(0.58, 0.68)
	(1)	BF protects baby from infection	0.14
	(2)	BF establishes close bond between mom and baby	0.18
	(3)	BF is embarrassing for mother	0.35
	(4)	BF is good for mother’s figure	−0.02
	(5)	BF limits a mother’s social life	0.24
	(6)	BF provides best nourishment for baby	0.25
	(7)	BF protects against infection	−0.76
	(8)	BF establishes close bond between mom and baby	−0.73
	(9)	BF does not make me feel embarrassed	−0.01
	(10)	BF is good for mother’s figure	−0.72
	(11)	BF allows social activity	−0.48
	(12)	BF provides complete nutrition	0.16
	(13)	FF is convenient feeding method	0.05
	(14)	FF makes it possible for baby’s father to be involved in feeding	0.16
	(15)	FF is expensive feeding method	0.27
	(16)	FF is trouble-free feeding method	−0.05
	(17)	FF allows one to see exactly how much milk baby has had	0.22
	(18)	FF provides incomplete nourishment for baby	0.19
	(19)	FF is convenient	−0.71
	(20)	FF allows for father’s involvement	−0.72
	(21)	FF is expensive	0.12
	(22)	FF is trouble free	−0.72
	(23)	FF allows one to see exactly how much milk baby has had	−0.57
Norms	(Q)	Response Options: Definitely Not (1) to Definitely (7)	(0.633, 0.72)
	(1)	BF Norms: baby’s father	0.42
	(2)	BF Norms: own mother	0.26
	(3)	BF Norms: closest female friend	0.42
	(4)	BF Norms: medical advisor	0.26
	(5)	FF Norms: baby’s father	0.83
	(6)	FF Norms: own mother	0.70
	(7)	FF Norms: closest female friend	0.73
	(8)	FF Norms: medical advisor	0.74
	(Q)	Response Options: Not at All Important (1) to Extremely Important (7)	
	(9)	Opinion of baby’s father	0.04
	(10)	Opinion of own mother	0.00
	(11)	Opinion of closest female friend	0.03
	(12)	Opinion of medical advisor	−0.09
	(Q)	Response Options: No (0), Yes (1)	
	(13)	Previous breastfeeding experience	0.00
Perceived Behavioral Control	(Q)	Response Options: Strongly Disagree (1) to Strongly Agree (7)	(0.34, 0.68)
	(1)	Perceived control over decision to breastfeed	0.33
	(2)	If I try my best I can breastfeed for 1 month	0.14
	(3)	If I try my best I can breastfeed for 3 months	0.87
	(4)	If I try my best I can breastfeed for 6 months	1.04
	(5)	Have tools/resources to breastfeed	0.64
	(6)	I have access to help in the event of breastfeeding problems	0.35
	(7)	Previous breastfeeding experience affected current decision to breastfeed	−0.11
	(8)	Previous breastfeeding experience painful	0.02
	(9)	Previous breastfeeding experience time consuming	−0.12
	(10)	Previous breastfeeding experience difficult	0.25
	(11)	Previous breast milk supply adequate to meet baby needs	0.11
Intentions	(Q)	*Response Options*: Strongly Disagree (1) to Strongly Agree (7)	(0.92, 0.91)
	(1)	Intend to exclusively breastfeed	0.82
	(2)	Intend to exclusively breastfeed at 1 month	0.755
	(3)	Intend to exclusively breastfeed at 3 months	0.976
	(4)	Intend to exclusively breastfeed at 6 months	0.874

*Note*: *FF* Formula Feeding, *BF* Breastfeeding

**Table 3:** Fit measures for the four latent measures

	CFI	TLI	RMSEA	SRMR
Attitude: First Order	0.37	0.307	0.154	0.147
Attitude: HLV	0.743	0.699	0.123	0.098
Norms: First Order	0.412	0.295	0.193	0.154
Norms: HLV	0.889	0.851	0.094	0.062
Perceived Behavioral Control: First Order	0.629	0.536	0.204	0.166
Perceived Behavioral Control: HLV	0.864	0.809	0.144	0.112
Intentions: First Order	0.942	0.826	0.248	0.033

Specifically, attitudes, subjective norms, and perceived behavioral control were not single factors, while breastfeeding intentions was a single factor (ω = 0.92 and α = 0.91). As such, the analysis progressed to exploratory factor analysis with a combination of a factor analysis with varimax rotation, and a MAP, VSS analysis, and parallels. This indicated that each domain within the TPB model was not a single factor, except for breastfeeding intentions. The feeding attitude factor was more accurately modeled as five factors, subjective norms as three factors, and perceived behavioral control as three factors. Additionally, the items that factored together did not always indicate a clear conceptual category. For example, the attitude item regarding expense factored with infection and nutrition (see Table 2 for question wording). This conceptual problem, however, did not apply to formula feeding items, which did cluster together quite well. That is, the questions related to formula feeding were statistically a single factor within the “attitude” domain (one of the five), but unlike other factors they also conceptually seem to belong with one another. Once new factor clusters were identified, analyses progressed to an attempt to fit a structural variable model approximating the TPB.

**Fig. 2:**
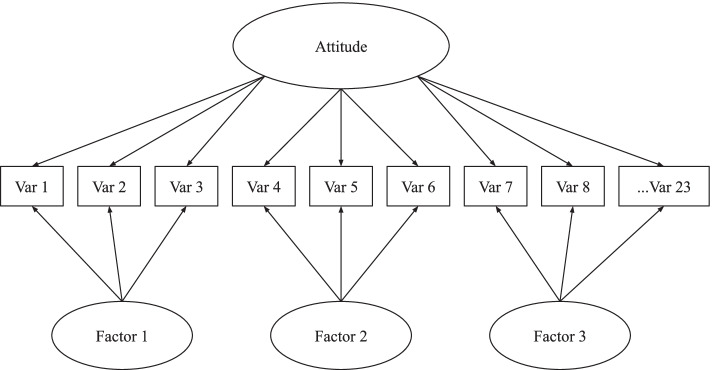
Bi-factor latent variable example for breastfeeding attitudes

The structural variable model approximating the TPB was attempted in four forms. First, a first order LV was specified (see Fig. 1). Reinforcing what is indicated by the ω and α scores (Table 2), only the Intentions domain was close to having an acceptable fit (Table 3, SRMR = 0.03, CFI = 0.94). Second, as many of the latent measures within the TPB model, beginning explicitly with attitudes, had multiple factors, the researchers attempted to create latent variables that still approximated the theoretic model with a bi-factor LV specification and then a hierarchical LV. The first of these was a bi-factor model (See Fig. 2), which uses a single latent general factor for the structural model that is composed of shared variance within the observed variables still present after first modeling domain-specific factor variance.

**Fig. 3:**
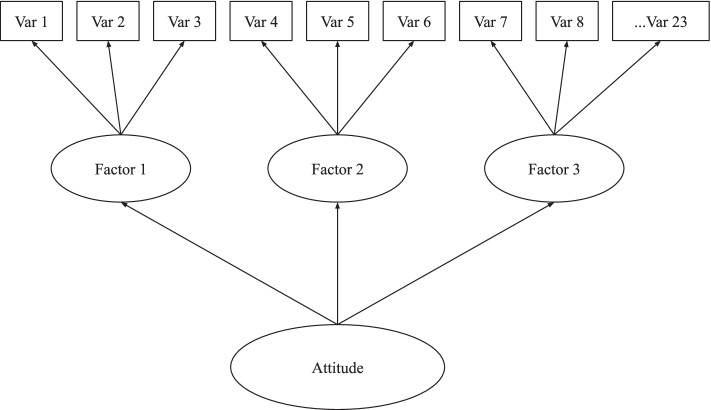
Hierarchical latent variable example for breastfeeding attitudes

For example, attitudes are composed of five factors. A bi-factor latent variable model first models the five factors and then attempts to model any remaining variance that could be in common amongst all observed variables as a general factor. None of these bi-factor LVs would converge, so there are no fit measures to include in Table 3. The second of the models approximating the TPB was a hierarchical latent variable model (See Fig. 3).

Similar to the bi-factor model, the hierarchical model first fits the subfactors (five for breastfeeding attitudes), but unlike the bi-factor model, it then fits the variance that is in common between these lower-order latent variables (as opposed to the observed variables as seen in the bi-factor model). The BIC did decrease with all of these HLVs, and many of the fit statistics also improved with this specification, but the best fit was with Norms, and this, while close, still did not hit acceptable model fit (RMSEA = 0.09 and SRMR = 0.06).

In a final attempt to fit a SEM approximating the TPB, analyses explored which of the factors from each domain of the TPB were most conceptually clear and best fit the overall theoretical argument of the model. Subsequently, just those factors (e.g. one factor from attitudes instead of all five) were included. The model fit indicated that the overall structure was not reliable and did not always converge. Thus, the reliability of even the significant paths is questionable.

## Discussion

This study attempted to use the TPB to explain variance in breastfeeding intentions and behaviors among a cohort of Midwest breastfeeding mothers. To do so, the research team used SEM to fit constructs within the TPB as latent variables and model breastfeeding behaviors. The constructs, however, did not hold up when modeled in this population. These issues with poor model fit may have gone unnoticed had the research team not opted to use SEM. This is especially apparent when considering construct alpha scores. The alpha scores for each construct with the TPB would have been acceptable, or nearly acceptable, for a traditional regression model (α > than 0.7). However, latent variables can parse measurement error more accurately. Alpha scores are based upon correlations among variables, but McDonald’s [19] omega score is based upon item factor loadings. In this way, unlike alpha, omega scores do not assume that all items contribute equally to constructs or that item errors are not correlated with one another [24]. When all observed variables measure the construct in the same way, as assumed in an alpha score, the omega score would be identical to an alpha [21]. In the case of the present data, the omega scores for the constructs are unacceptably lower than the alpha scores, indicating that they are less reliable than would otherwise be known (see Table 2).

Unlike Duckett and colleagues [4] and Dodgson and colleagues [25], the TPB did not fit these data. This difference may not mean the TPB is an entirely invalid model, but it does indicate that there are problems that question the reliability in its present form. The authors’ primary reasoning for this difference could be related to current SEM methods since statistical packages and computing power have advanced. Additionally, Dodgson and colleagues [25] appeared first to create summed scales and then produced their SEM with the resulting correlation matrix. The problem, however, arises before the point of creating additive scales. This method masks the fact that the items in the scale do not belong together. This finding can go unnoticed as alpha scores can border on acceptable, but using an omega score that does not rely on the same assumptions flags problems. Similar to the findings in this study, Dodgson and colleagues’ alpha score on control was their lowest, although in their case alpha scores were higher and within more acceptable ranges. In our case, this is notable because we were also within an acceptable alpha range (α=0.68) but the omega flags problems with a score of only 0.34, which is very low. In other words, the problems highlighted here have more to do with the items theorized to belong together within each domain. Yet, the authors remain agnostic regarding the possible overall theorized relationships as they may remain effective.

Guo and colleague’s [7] meta-analysis is a welcome addition to breastfeeding behavior research as understood through the TPB. However, the results rely heavily on published correlation matrices that make use of additive scales that implicitly and statistically assume equal item variance within construct domains. The selected methods inadvertently obfuscate how poorly the unreliable instrument fits the lived experience, confirming the need to refine and update the instrument.

For some time, research focused on the formation of beliefs and values and the actions that flow from socialization has highlighted the importance of close ties. Exposure to relationships, social structures, and one’s position within them, shape perceptions of the world that are then replicated in behaviors [26, 27]. Typical Midwest family structures and relational ties within the Midwest, and this location, specifically, likely have a large influence on mothers’ perceptions of default desirable behaviors, including breastfeeding behaviors.

Study participants were primarily recruited via social media (Facebook), indicating this cohort of childbearing women is active on social media and, at least in this case, social media activity is connected to actions. Recent research has shown that women are more active in seeking out health information than men and that the internet plays a key role in this information consumption [28]. This idea holds true to seeking specific information tied to breastfeeding [29–32]. As such, the content and slant of breastfeeding information presented on social media is likely a key unmeasured variable for the patterning of breastfeeding behaviors. However, the questionnaires utilized in this study failed to gather data related to the influence of social media and social networking sites on the attitudes, subjective norms/normative beliefs, perceived behavioral control, and intentions toward breastfeeding behaviors.

While the overall efforts to fit a SEM for the TPB failed, variables related to formula feeding clustered together quite well in this study**.** Beyond the fact that formula measures factoring well is conceptually notable, this may also be a small indication that the failure of TPB to fit this data well is not merely a data artifact. After all, it would not make sense to say it is merely a data problem when some manifest measures that clearly belong together (formula feeding questions) statistically do hold together quite well. If there were a fundamental problem with the data, it would be odd for it to not be widespread throughout the measures. The uniformity of messaging by the formula industry may explain why those items may more clearly factor out together. Strong, uniform messaging is also a key reason why mothers may discontinue breastfeeding [33]. Although formula marketing is down in the US, it may be that there remains a long-standing impact on formula feeding attitudes. In contrast, breastfeeding messaging remains uncoordinated and unclear, even among health care providers [34, 35].

### Limitations

The limitations of this study include convenience sampling, a limited sample size, homogeneity of the study sample, and the use of previously developed questionnaires with a limited ability to capture the constructs under investigation. Despite these limitations and null findings, this study remains particularly valuable to nursing science, in which the development of interventions is driven by theory. Among dissertations focused on breastfeeding research during the last 10 years, approximately eight dissertations utilized the TPB as a guiding theory. This does not encompass the multitude of studies in maternal and child health currently underway, nor published manuscripts using the TPB as a guiding framework.

## Conclusion

This research highlights limitations in tools developed to measure the TPB theoretical constructs of attitudes, subjective norms/normative beliefs, perceived behavioral control, and intentions related to breastfeeding behavior. Despite the fact that data in this study are composed of a relatively homogeneous sample of mothers from the same community, attempts to fit an SEM failed. The research team speculates that these deficits may be related to the use of outdated tools lacking cultural relevance, a change in social norms, and a failure to capture the possible influence of social media and formula marketing on breastfeeding behaviors.

In addition, this study demonstrates the importance of using methods that fit the phenomena explained. The research team used SEM to fit constructs within the TPB as latent variables and model breastfeeding behaviors, which did not hold up when modeled. These issues with poor model fit may have gone unnoticed had the research team not opted to use SEM. As a result, the present null finding is a significant finding indicating the need to revisit and refine the operationalization and conceptual underpinnings of the TPB through qualitative methods. This would include exploring the lived experiences of breastfeeding women in pregnancy and during lactation, taking into account breastfeeding difficulties (nipple/breast pain, poor latch, ineffective suck) or maternal (insufficient glandular tissue, delayed or impaired lactogenesis, etc) and infant factors (late preterm, ankyloglossia, hyperbilirubinemia, etc.) that may have a significant impact on women’s decision or ability to continue breastfeeding in the Midwest region.

## Data Availability

Participant consent was not obtained to make the data publicly available. Requests for data can be made to the corresponding author.

## Abbreviations

TPB: Theory of Planned Behavior
MAP: Minimum Average Partial
VSS: Very Simple Structure
SEM: Structural Equation Models
LV: Latent Variable
BIC: Bayesian Information Criterion
CFI: Comparative Fit Index
TLI: Tucker-Lewis Index
RMSEA: Root Mean Squared Error of Approximation
SRMR: Standardized Root Mean Squared Residual
